# The roles of kinetochore of micronucleus in mitosis of HeLa cells: a live cell imaging study

**DOI:** 10.1186/s12935-019-0917-8

**Published:** 2019-08-02

**Authors:** Erkang Jiang, Lianping Wei, Fang Tao, Mei Yu, Shu Wang, Xiuhong Zhou, Daxiang Li, Zhongwen Xie

**Affiliations:** 1 State Key Laboratory of Tea Plant Biology and Utilization, Anhui Agricultural Universiy, Hefei, 230036 Anhui People’s Republic of China; 2 School of Life Sciences, Anhui Agricultural Universiy, Hefei, 230036 Anhui People’s Republic of China

**Keywords:** Micronucleus, Kinetochore, Lagging chromosome, Chromosome fragment, Mitosis, Live cell imaging

## Abstract

**Background:**

Micronuclei (MNi) are extensively used to evaluate genotoxic effects and chromosome instability. However, the roles of kinetochore of MN in mitosis have not been completely addressed.

**Methods:**

The HeLa CENP B-GFP H2B-mCherry cells are applied to address these questions via the long-term live-cell imaging. In the cells, the kinetochore-positive micronucleus (K+MN) contained CENP B-GFP, while the kinetochore-negative micronucleus (K−MN) did not.

**Results:**

K−MN-bearing cells produced much more chromosome fragments than did MN-free cells. Most of the chromosome fragments eventually merged into K−MNi. K+MN-bearing cells yielded more kinetochore-positive lagging chromosomes (K+LCs) and K+MNi than MN-free cells did. The results suggested the differences in the fates of K+MNi and K−MNi in mitosis. The cycle of K−MN → Chromosome fragment → K−MN may occur in generations of K−MN-bearing cells, while part of K+MNi might reincorporate into the main nucleus. The K+MN-bearing cells prolonged significantly duration of mitosis compared with MN-free cells. The presence of micronuclei, regardless of K−MN and K+MN, enhanced apoptosis cell death. And K+MN-bearing cells were inclined to apoptosis more than K−MN-bearing cells. The results suggested differences in fates between K−MN-bearing and K+MN-bearing cells.

**Conclusions:**

Kinetochore determined the fates of micronuclei. Kinetochore in micronuclei indirectly prolonged the duration of mitosis. Kinetochore enhanced cytotoxicity of micronuclei. Our data are direct evidences showing the roles of kinetochore of micronucleus in mitosis of HeLa cells.

**Electronic supplementary material:**

The online version of this article (10.1186/s12935-019-0917-8) contains supplementary material, which is available to authorized users.

## Background

The micronucleus (MN) test determines chromosomal level DNA damage and is widely used to biomonitor humans exposed to clastogens and aneugens [1, 2]. Elevated frequencies of MNi are also found in patients with cancer and other diseases [3, 4]. MNi are formed from an entire chromosome or from a chromosomal fragment. The kinetochore is an essential structure composed of a number of conserved protein complexes on the centromere in eukaryotes. It serves as a bridge between the spindle microtubules and chromosomes and regulates chromosome segregation [5, 6]. Based on the presence of kinetochores, MNi are further classified into K+MNi and K−MNi. In fixed cells, kinetochores in MNi can be detected by immunofluorescent staining using anti-kinetochore antibodies from the serum of scleroderma (CREST syndrome) patients. Aneugenic agents mainly induce K+MNi in human cells, while clastogenic agents enhance K−MNi. The classification increases the specificity of the MN test [7–11].

In live cells, kinetochores in MNi were identified in a dual-colour fluorescent cell line, HeLa CENP B-GFP H2B-mCherry cells [12]. In these cells, chromosomes and kinetochores were labelled by H2B-mCherry and CENP B-GFP, respectively. MNi were marked by H2B-mCherry. K+MNi were identified by CENP B-GFP, while K−MNi did not have the GFP signal. The differences in the origins of K+MNi and K−MNi were investigated using this construction [12]. However, the roles of kinetochore of micronucleus in mitosis of HeLa cells have not been completely addressed.

Dynamic MN formation was analysed in several types of living cells [13–15]. The MN-bearing cells frequently produced daughter cells with MNi through chromosome lagging during cell division [16]. MNi were partly reincorporated into daughter nuclei after mitosis [17]. If this is the case, there should be significant differences between cells with K+MNi and K−MNi, because K+MNi contain kinetochore structures and K−MNi not. When K+MN-bearing cells enter mitosis, the chromosomes from K+MNi may be indistinguishable from those of the main nucleus and might resume normal biological activity. While K−MN-bearing cells enter mitosis, the chromosomal fragments in K−MNi cannot be caught by spindle microtubules because they do not have functional kinetochores and subsequently fail to be pulled onto the metaphase plate. Chromosomal material from K−MNi may condense into chromosome fragments in meta-anaphase and might reform as K−MNi in daughter cells. In other words, K−MN-bearing cells may produce more chromosome fragments and K−MNi than do MN-free cells during mitosis. K+MN-bearing cells might form more K+LCs and K+MNi during cell division but to a lesser extent, because some of the chromosomes from K+MNi may reincorporate in the main nucleus.

To test this possibility, multi-layer high-resolution imaging was conducted by using HeLa CENP B-GFP H2B-mCherry cells. The dynamics of mitosis in K+MN- and K−MN-bearing cells were accurately recorded over short intervals during mitosis. The fates of K−MNi and K+MNi in mitosis were investigated by reverse examination of these time-lapse records, as well as the fates of K−MN- and K+MN-bearing cells.

## Methods

### Cell culture

HeLa CENP B-GFP H2B-mCherry dual-color fluorescent cells were constructed in our laboratory [12] and cultured in DMEM supplemented with 10% fetal calf serum, 1% non-essential amino acids and antibiotics (100 U/mL penicillin and 100 U/mL streptomycin). These regents were purchased from GIBCO. The cell cultures were maintained at 37 °C in a humidified atmosphere of 5% CO_2_ and 95% air.

### Live cell imaging

HeLa CENP B-GFP H2B-mCherry cells were grown on coverglass-bottomed dishes (MatTek, USA) at a density of 2 × 10^5^ cells per dish for 26 h. Live-cell imaging was performed as previously described [12]. In order to detect abnormal chromosome and MN as possible, ten-layer imaging was performed on each cell by fluorescent microscope.

### Analysis of the live cell images

The image series were obtained from the live cell imaging experiments and then were converted into movies using the Metamorph software (Universal Imaging Corporation, USA). The following criteria were applied to distinguish the abnormalities, stages and polar number in mitoses during the scoring. The definition of MNi, Kinetochore-positive lagging chromosomes (K+LCs) and Chromosome bridges was described in the previous study [12]. Chromosome fragment and Stage of mitosis were described in Additional file 1: Methods.

### Statistical analysis

The data were analyzed using the 2 × 2 Chi square test. A *p*-value of less than 0.05 was statistically significant and a *p*-value of less than 0.001 was highly significant.

## Results

**Fig. 1:**
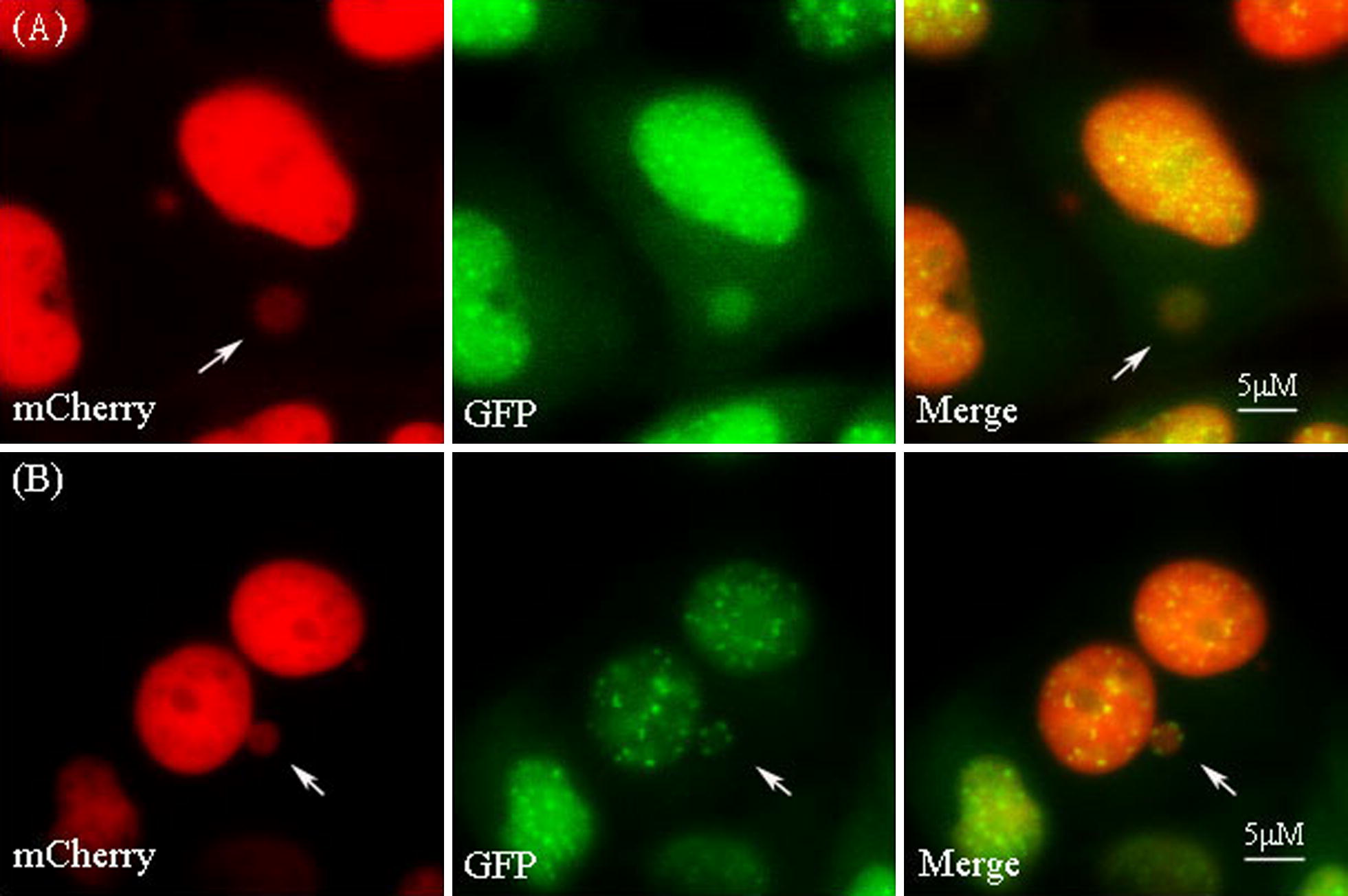
Representative figures showed that a K−MN and K+MN were classified by CENP B-GFP in HeLa CENP B-GFP H2B-mCherry cells. Cell imaging was performed by using an inverted fluorescent microscope. In the dual-color fluorescent cell line, micronuclei (MNi) were labelled by H2B-mCherry. A K+MN carried a CENP B-GFP signal, while K−MN did not. Selected serial images (including mCherry, GFP and merged images) from time-lapse records by show examples of: **A** A representative figure of a K−MN. An arrow points to a K−MN carrying the mCherry signal. **B** A representative figure of a K+MN. An arrow points to a K+MN carrying both mCherry and GFP signals

In present study, HeLa cells were scored by examining the long-term real-time images, which included different types of mononuclear cells, i.e. MN-free cells (micronucleus-free cells), K−MN-bearing cells (cells each bearing a K−MN) and K+MN-bearing cells (cells each bearing a K+MN) (Fig. 1). After these cells entered mitosis, dynamic of mitosis was recorded and analyzed. Emergences and fates of aberrant chromosome during mitosis were observed in different type cells. And we investigated duration of mitosis and apoptosis in these three types of cells.

### Emergence of aberrant chromosomes in different type cells

**Fig. 2:**
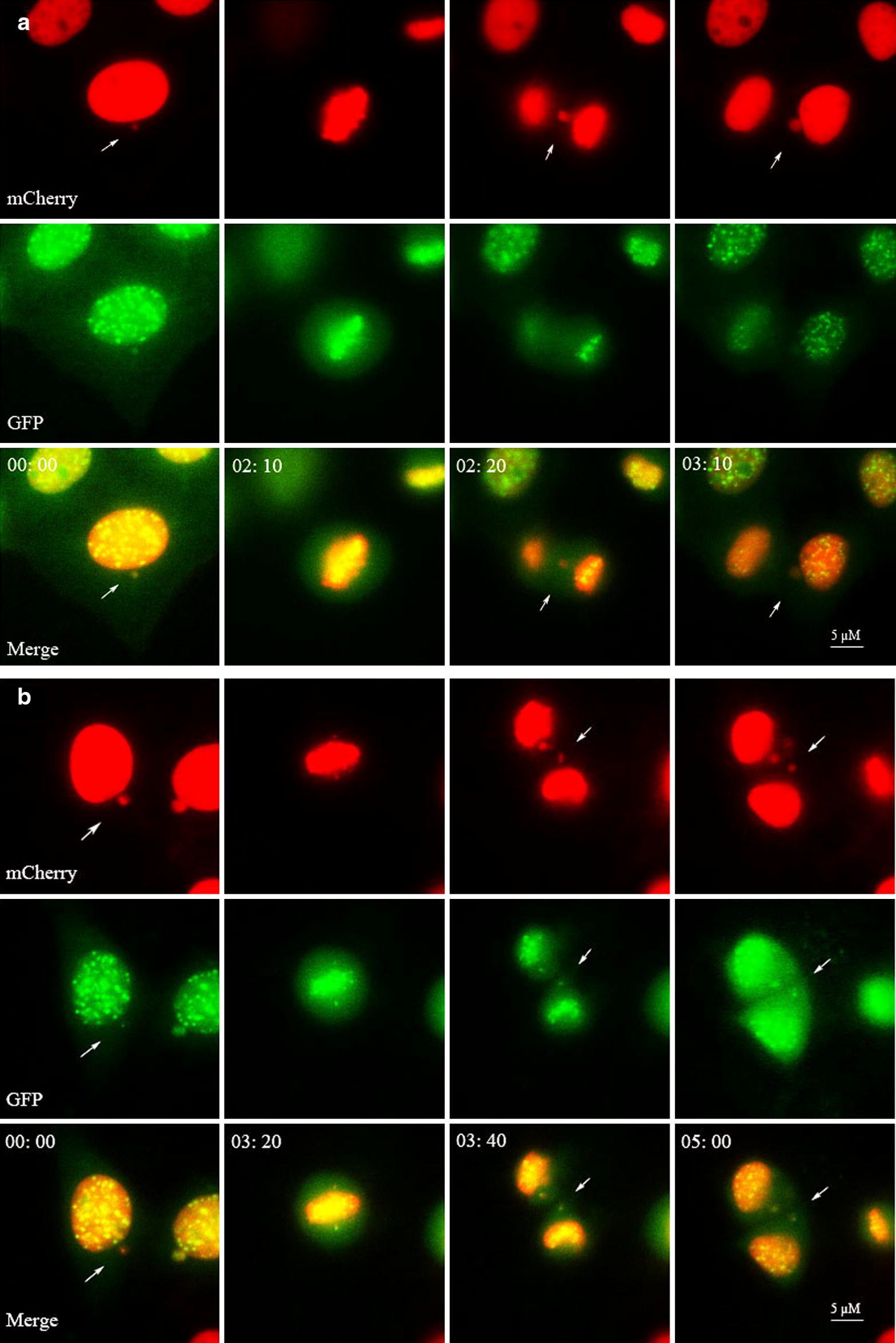
Representative figures of chromosome fragment and K+LC emerged during mitoses of K−MN-bearing and K+MN-bearing cells. In the HeLa CENP B-GFP H2B-mCherry cells, selected serial images (including mCherry, GFP and merged images) from time-lapse records show examples of: **a** A K−MN-bearing cell produced a chromosome fragment during anaphase and a K−MN in a daughter cell. Arrows points to a mother cell, a chromosome fragment and a K−MN, sequentially. **b** A K+MN-bearing cell produced a K+LC during anaphase and a K−MN in a daughter cell. Arrows points to a mother cell, a K+LC and a K+MN, sequentially

**Fig. 3:**
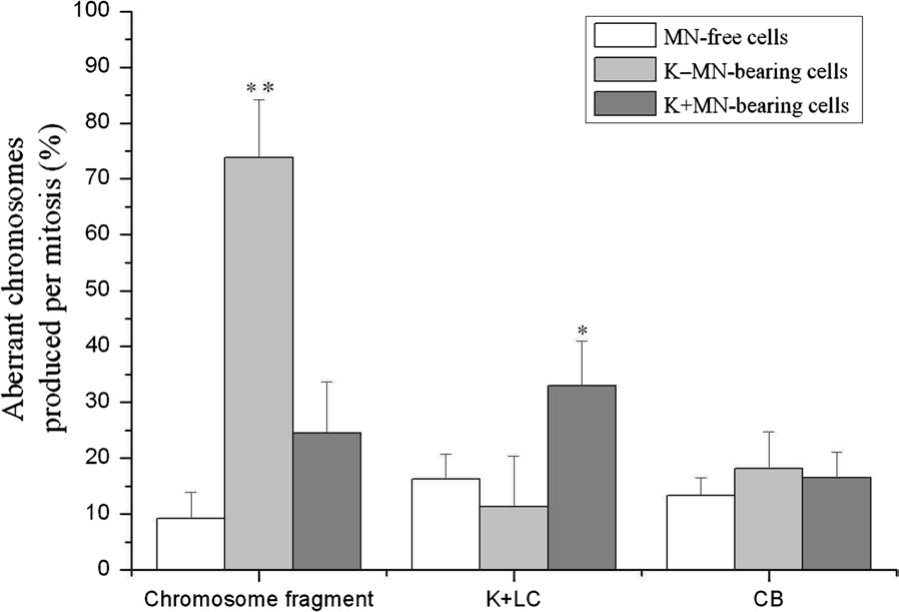
K−MN- and K+MN-bearing cells incline to produce special abnormal chromosome in mitosis with different frequencies. Nuclear and cell divisions with chromosomal segregation anomalies were followed to determine that the relationship between K−MN or K+MN and abnormal chromosome in mitosis of MN-bearing cells. Chromosome fragments carried only H2B-mCherry signals, but no CENP B-GFP. K+LCs emerged in anaphase and carried both H2B-mCherry and CENP B-GFP signals. CBs were identified using H2B-mCherry signals, regardless of the CENP B-GFP status. K−MN-bearing cells produced chromosome fragments much more than MN-free cells in mitosis, while K+MN-bearing cells produced significantly K+LCs more than MN-free cells (*p < 0.05, **p < 0.01, compared with MN-free cells. n = 3)

In this study, the dynamics of aberrant chromosomes were observed through live cell imaging in the HeLa cells. Frequencies of aberrant chromosomes produced by different type of cells in mitosis are variable (Figs. 2, 3). The K−MN-bearing cells produced much higher frequency of chromosome fragment (73.69 ± 14.88%) than that (9.19 ± 4.66%) of MN-free cells; and than that (24.46 ± 9.27%) of K+MN-bearing cells during mitosis. The K+MN-bearing cells yielded the higher rate of K+LC (32.97 ± 8.0%) than that (16.29 ± 4.39%) of MN-free cells and that (11.29 ± 9.09%) of K−MN-bearing cells during mitosis. Furthermore, the CBs were identified using H2B-mCherry signals, regardless of the CENP B-GFP status. The MN-free cells produced slightly less frequency of CB (13.35 ± 3.17%) than that (18.12 ± 6.68%) of K−MN-bearing cells and that (16.52 ± 4.55%) of K+MN-bearing cells during mitosis, but did not reach significance. So, different type cells produced different aberrant chromosomes with different frequencies in mitosis.

### Formation of K−MN and K+MN in different cells

**Fig. 4:**
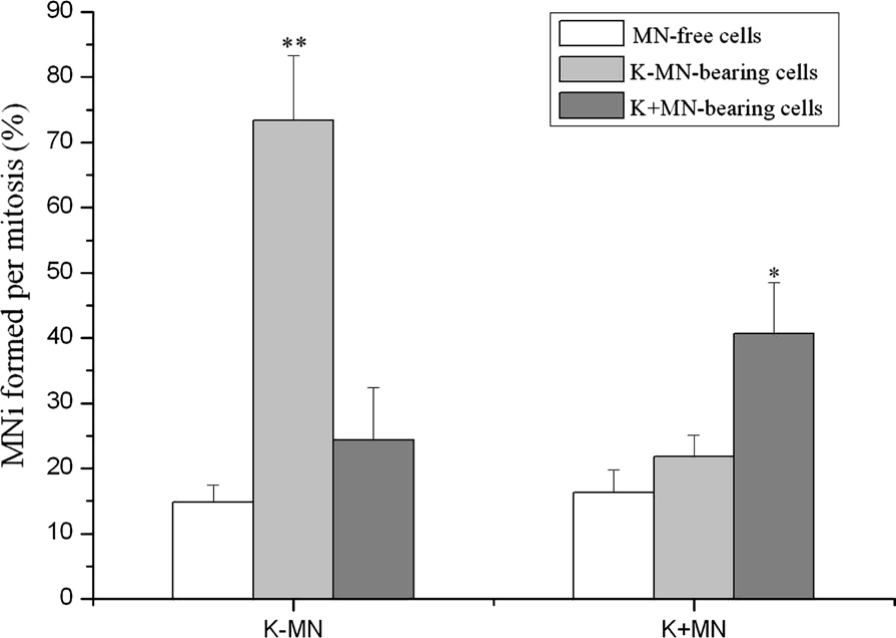
MN-bearing cells are prone to produce the same kind of MN in daughter cells with different frequencies. MN-bearing cells were followed during mitosis to determine that the relationship of MNi between two generations. K+MNi carried both H2B-mCherry and CENP B-GFP signals. K−MNi contained only H2B-mCherry signals, but no CENP B-GFP. K+MN-bearing cells produced significantly more K+MNi than MN-free cells in daughter cells, while K−MN-bearing cells formed K−MNi in much more frequencies. (*p < 0.05, **p < 0.01, compared with MN-free cells. n = 3)

The frequency of K−MN formation (73.33 ± 10.03%) during mitosis of K−MN-bearing cells was much higher than that (14.78 ± 2.73%) in MN-free cells and that (24.39 ± 8.05%) in K+MN-bearing cells (Fig. 4). The rate of K+MN formation (40.69 ± 7.77%) in mitosis of K+MN-bearing cells was significantly higher than that (16.34 ± 3.48%) in MN-free cells and than that (21.86 ± 3.25%) in K−MN-bearing cells. So, K−MN-bearing cells produced much more K−MNi in daughter cells than MN-free cells. Similarly, K+MN-bearing cells generated more K+MNi in daughter cells than MN-free cells, but in a less frequency.

### Durations of mitosis were prolonged in K+MN-bearing cells

**Fig. 5:**
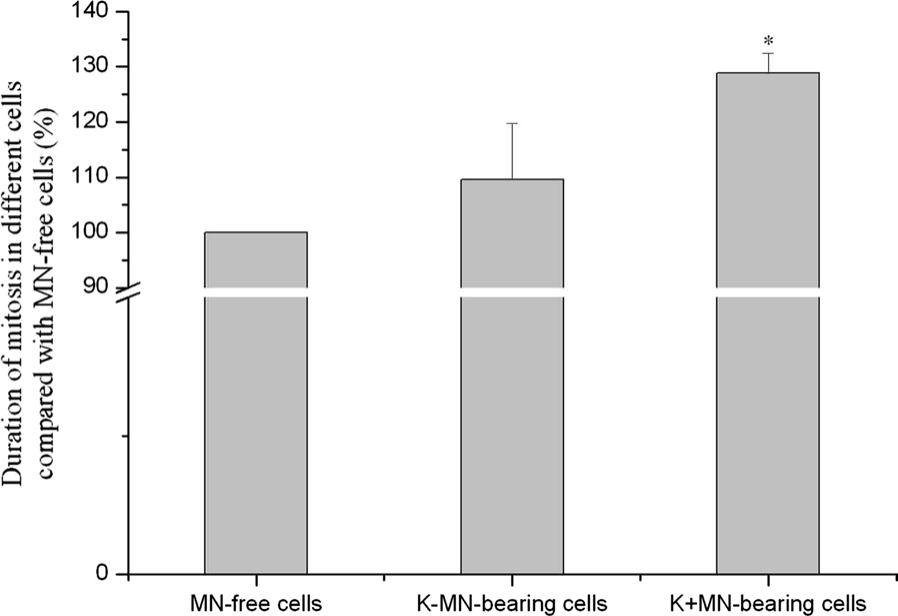
The duration in mitosis of K+MN-bearing cells is longer significantly than that in MN-free cells. During the time-lapse observation, the four stages—prophase, metaphase, anaphase, and telophase were followed to determine the relationship between the presence of MN and the duration of mitosis in HeLa CENP B-GFP H2B-mCherry cells. The presence of K+MN prolonged obviously the duration of cell mitosis (*p < 0.05, compared with MN-free cells. n = 3)

Mitosis is conventionally divided into four stages—prophase, metaphase, anaphase, and telophase in an animal cell. During the time-lapse observation, the time points of the four stages of mitosis were recorded and analyzed (Additional file 2: Figure S1). To our surprise, durations of mitosis were different in three types of cells (Fig. 5). The durations from prophase to telophase in K+MN-bearing cells were significantly longer and increased by 28.8% than that in MN-free cells. The durations from prophase to telophase in K−MN-bearing cells were slightly longer than that in MN-free cells, but did not reach significance. Our results indicated that the duration of mitosis in K+MN-bearing cells is significantly longer than that in MN-free cells.

### Fates of K−MN-bearing cells and K+MN-bearing cells

**Fig. 6:**
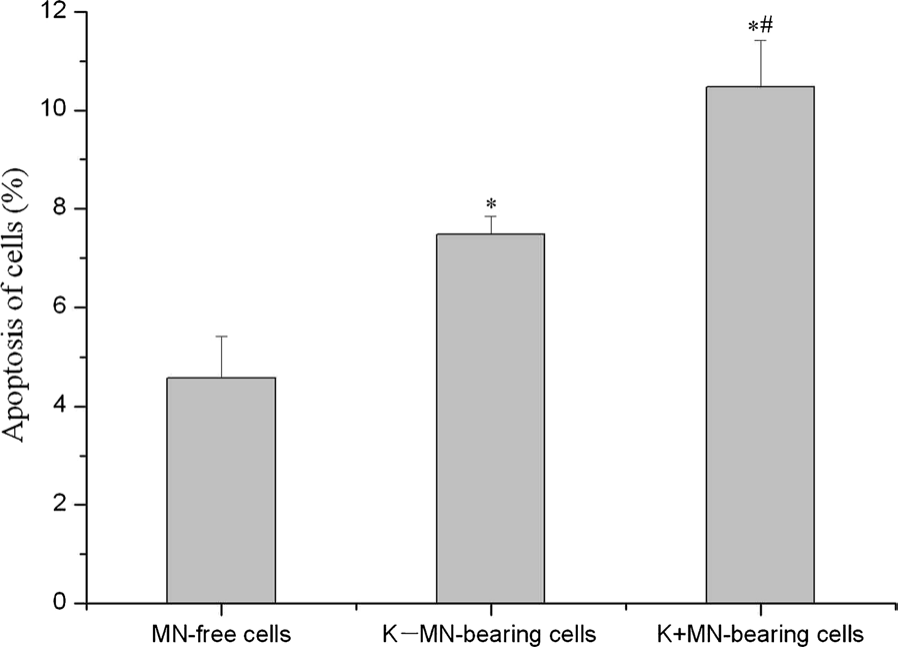
Frequencies of apoptosis K+MN-bearing cells were significantly higher than that in K−MN-bearing and MN-free cells. During the time-lapse observation, fates of MN-bearing cells were followed to determine the relationship between the presence of MN and apoptosis. K−MN increased markedly apoptosis of cell, while K+MN did in the most frequency. (*p < 0.05, compared with MN-free cells; ^#^p < 0.05, compared with K−MN-bearing cells. n = 3)

During the course of time-lapse observation, we found that the frequencies of apoptosis were different in different cells (Fig. 6 and Additional file 3: Figure S2). The frequencies of apoptosis in K+MN-bearing cells (10.47 ± 0.95%) and K−MN-bearing cells (7.48 ± 0.37%) are both significantly higher than in MN-free cells (4.58 ± 0.84%). The result is consistent to previous studies. For the more, the frequency of apoptosis in K+MN-bearing cells is markedly higher compared with K−MN-bearing cells.

If the cells bear more MNi, the frequency of apoptosis becomes higher. The frequency of apoptosis in cells with 2K−MNi was 30%, and the frequency of apoptosis in cells with 2K+MNi was 40%. So, Presence of K−MNi or K+MNi were frequently associated with apoptosis, rather than multipolar mitosis (data not shown).

## Discussion

In a previous study by our laboratory, the HeLa CENP B-GFP H2B-mCherry cell line was constructed [12], in which K+MNi were reliably distinguished from K−MNi by CENP B-GFP signals in living cells. The cell line can be applied in the study of the genotoxic effects of anticancer drugs (Additional file 4: Table S1), which is crucial for understanding the mechanism of their activity in the possible generation of secondary tumors. And by using the cell line, the emergence of abnormal nucleic structures was investigated [12].

K−MNi originate mainly from chromosome fragments and CBs. K+MNi derive predominantly from K+LCs and CBs. Several questions remain to be answered, such as: are the processes of MN formation reversible in the next cell division? Can K−MNi transform into chromosome fragments and CBs, or K+MNi into K+LCs and CBs, in mitosis?

The frequency of CB was not enhanced in mitosis of K−MN- and K+MN-bearing cells compared to MN-free cells. The data suggested that K−MNi and K+MNi were unlikely transformed into CBs in mitosis, although CBs were one of main origins of K−MNi and K+MNi [12, 13].

A recent study showed that chromosomes within MNi reincorporated into daughter nuclei at a significant frequency during mitosis [18, 19]. In the study, K+MN-bearing cells produced more K+LCs and K+MNs during mitosis than MN-free cells. The results suggested that part of K+MNi may transform into K+LCs and K+MNi. As there are spontaneous K+LCs and K+MNi in mitosis of MN-free HeLa cells, not all K+LCs or K+MNi resulted from K+MNi of mother cells. However, it is not likely that all K+MNi reincorporated into daughter nuclei, for defects of MN structure [19]. Subsequently, part of chromosomes in K+MNi may be able to reincorporate into main nuclei.

In contrast to K+MNi, if chromosomal materials in K−MNi condense into chromosomal fragments during prophase, they are unlikely to return to main nuclei and frequently evolve into K−MNi in daughter cells. The results in the study showed that K−MN-bearing cells produced much more chromosomal fragments in mitosis and K−MNs in daughter cells than MN-free cells. The data indicated that the cycle of K−MN → Chromosome fragment → K−MN might occur during mitosis.

If this is the case, the more K−MNi cells bear, the more chromosome fragments and K−MNi emerge during mitosis and in daughter cells, respectively. In this study, there were five cells, each of which contained two K−MNi. They produced eight chromosome fragments and transformed into seven K−MNi in six daughter cells (data not shown). What will happen if a cell bears K−MN and K+MN, instantaneously? There were four cells which each carried both a K−MN and a K+MN; these produced four chromosome fragments and one K+LC in mitosis, which further evolved into four K−MNi and one K+MN in daughter cells (data not shown). These results show different fates between K−MN and K+MN in the same cell and kinetochores determine the fates of MNi.

**Fig. 7:**
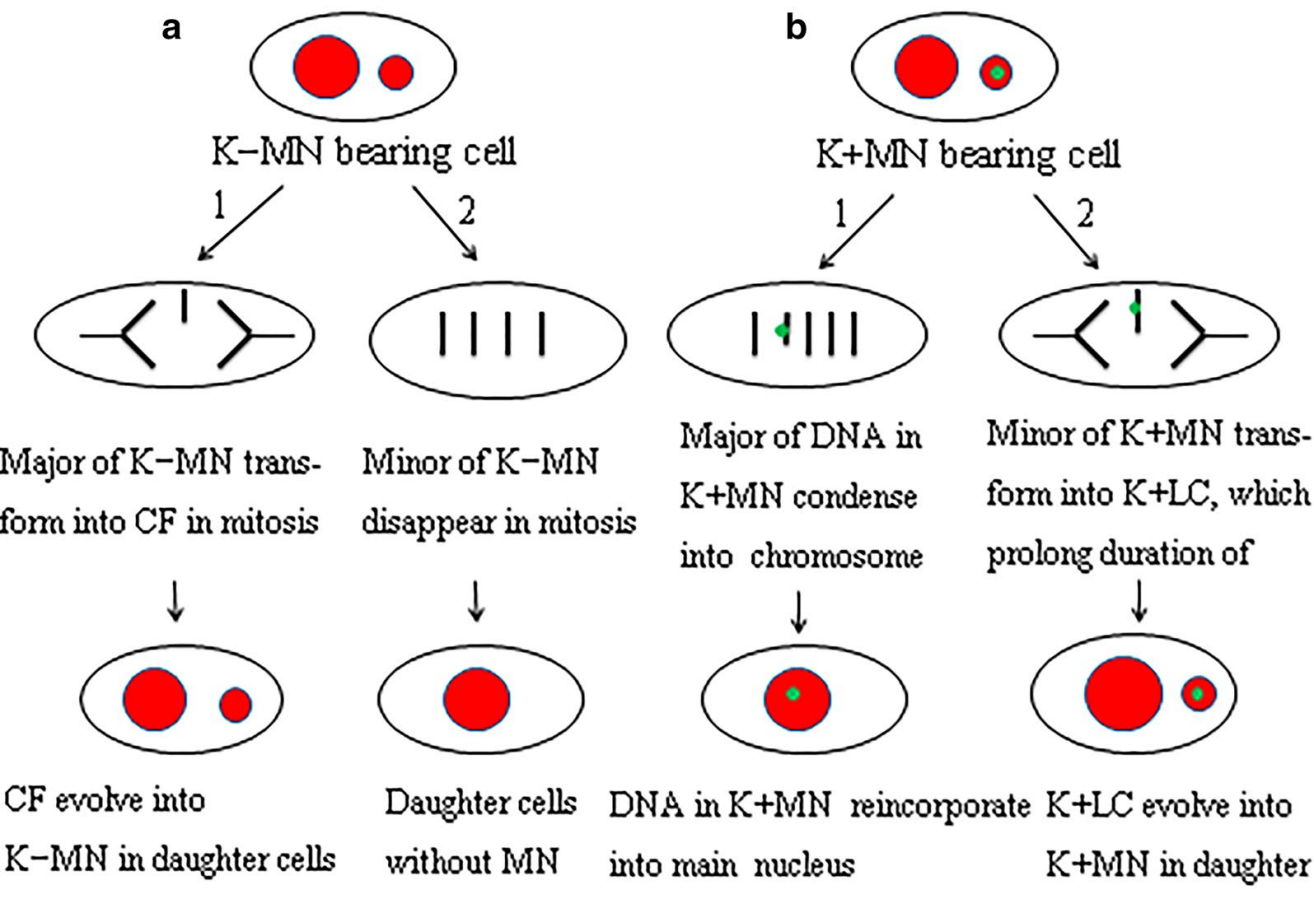
Final models show differences in fates between K−MN and K+MN in mitois. Based on the study, fates of of K−MN and K+MN were included in final models.** a** The fates of K−MN in mitosis. (1) The cycle of K−MN → CF (Chromosome fragment) → K−MN. If K−MN-bearing cells enter mitosis, major of K−MNi transform into CFs, which CFs evolve into K−MNi in daughter cells. (2) Disappearance. When K−MN-bearing cells enter mitosis, minor of K−MNi disappear in mitosis.** b** The fates of K+MN in mitosis. (1) Reincorporation. If K+MN-bearing cells enter mitosis, major of K+MNi reincorporate into main nuclei and no K+MNi emerge in daughter cells. (2) The cycle of K+MN → K+LC → K+MN. Minor of K+MNi transform into K+LCs, which evolve into K+MNi in daughter cells. The models show differences in fates between K−MN and K+MN. And kinetochores determine the fates of K−MN and K+MN in mitosis

If there were displaced chromosome in metaphase, the spindle assembly checkpoint (SAC) would delay anaphase until all chromosomes are properly aligned at the spindle equator [20]. The K+MN-bearing cells produced more K+LCs during mitosis than that of MN-free cells. Many K+LCs in anaphase results from displaced chromosomes [12]. So the results might provide an explanation for prolonging duration of mitosis in K+MN-bearing cells. On the country, K−MN-bearing cells slightly prolonged the durations of mitosis than that in MN-free cells, although emergence of much more chromosome fragments during mitosis. Based on the study, fates of of K−MN and K+MN in mitosis were included in final models (Fig. 7).

We further investigated whether K−MNi or K+MNi affect fates of cells, or not. Our data indicated that the presence of micronuclei, including K−MN and K+MN, enhanced apoptosis cell death. It is consistent to the previous study. And we further proved that K+MN-bearing cells were inclined to apoptosis more than K−MN-bearing cells. The results suggested differences in fates between K−MN-bearing and K+MN-bearing cells.

Since presence of K−MN or K+MN can enhance apoptosis of cell, we want to know whether K−MN or K+MN increase degradation of genomic DNA. Experiments for DNA degradation were performed to compare the degradation level in genomic DNA which contained mainly K−MNs or K+MNs (Additional file 5: Figure S3). Results indicated that DNA degradation did not occur obviously in K−MN- or K+MN-containing genomic DNA. K−MN- and K+MN-bearing cells could not be separated and collected perfectly in the test. In the future study, we would find appropriate means to investigate the genomic DNA degradation of K−MN- and K+MN-bearing cells.

## Conclusions

This study aimed to investigate the roles of kinetochore of micronucleus in fates of K−MNi and K+MNi, as well as in fates of K−MN- and K+MN-bearing cells. The results can be briefly summarized in the following points.

First, kinetochore determined the fates of micronuclei in mitosis. The chromosomes in K+MNi might reincorporate into the main nucleus, while K−MNi may be involved in the cycle of K−MN → chromosome fragment → K−MN.

Second, kinetochore in micronuclei indirectly prolonged the duration of mitosis. K+MNi may transform into K+LCs in mitosis, which resulted in longer duration of mitosis. In the contrary, K−MNs did not enhance the duration of mitosis, although K−MNs transformed into chromosome fragments in much more frequency during mitosis.

Third, kinetochore enhanced cytotoxicity of micronuclei. Presence of micronuclei was frequently associated with apoptosis. And K+MN-bearing cells were more inclined to apoptosis than K−MN-bearing cells. Subsequently, K+MN showed cytotoxicity more than K−MN.

These direct evidences show functional roles of kinetochore in micronuclei during mitosis of HeLa cells.

## Additional files

**Additional file 1.** Methods.

**Additional file 2: Figure S1.** Representative figure for duration of bipolar mitosis in a MN-free HeLa CENP B-GFP H2B-mCherry cell. Selected serial images (including mCherry, GFP and merged images) from time-lapse records showed four stage of mitosis. a. Prophase, the beginning of prophase is marked by the appearance of condensed chromosomes. b. Metaphase, the chromosomes align in the centre of the spindle, or the equatorial plate. c. Anaphase, the sister chromatids separate and move to opposite poles of the spindle. d. Telophase, the sister chromatids reach opposite poles and de-condense.

**Additional file 3: Figure S2.** Representative figures for apoptosis of a MN-free HeLa CENP B-GFP H2B-mCherry cell. Selected serial images (including mCherry, GFP and merged images) from time-lapse records showed apoptosis of a cell in mitosis. Arrows point to the initial cell nucleus, its pyknosis and Karyorrhexis.

**Additional file 4: Table S1.** Colcemid and actinomycin D induced K−MNi and K+MNi in HeLa CENP B-GFP H2B-mCherry cells.

**Additional file 5: Figure S3.** DNA degradation was not obvious in Hela Cells containing mainly K**−**MNs and K**+**MNs. Cells were exposed to actinomycin D and colcemid and for 24 h, and DNA were isolated from each treatment for gel electrophoresis as described in “Methods” section. (1) 100 bp DNA ladder marker (Takara Corp.); (2) Control; (3) Cells treated with 150 ng/mL actinomycin D; (4) Cells treated with 15 ng/mL actinomycin D; (5) Cells treated with 25 ng/mL colcemid. Results suggested that there was no DNA degradation in control cells. DNA degradation was obvious in the high concentration of actinomycin D treatment (150 ng/mL), while slight DNA degradation occurred in the colcemid and low concentration actinomycin D (15 ng/mL) treatment cells.

## Data Availability

Not applicable.

## Abbreviations

MN: micronucleus
MNi: micronuclei
K−MN: kinetochore-negative micronucleus
K+MN: kinetochore-positive micronucleus
LC: lagging chromosome
K+LC: kinetochore-positive lagging chromosome
CB: chromosome bridge
