# Supplementary material for: The roles of kinetochore of micronucleus in mitosis of HeLa cells: a live cell imaging study
Source: Cancer Cell Int. 2019 Aug 2;19:206. doi: 10.1186/s12935-019-0917-8 (PMC6679434; doi:10.1186/s12935-019-0917-8)
Supplement: Supplementary file 1 — Additional file 1. Methods. [file 12935_2019_917_MOESM1_ESM.docx]

**Methods**

**Chromosome fragments** Chromosome fragment are kinetochore-negative pieces of chromosomes, which contain only H2B-mCherry signals, but no CENP B-GFP.

**Stages of mitosis** Four stages of bi-mitosis were recorded during live cell imaging. First, prophase, the beginning of prophase is marked by the appearance of condensed chromosomes. Second, metaphase, the chromosomes align in the centre of the spindle, or the equatorial plate. Third, anaphase, the sister chromatids separate and move to opposite poles of the spindle. Forth, telophase, the sister chromatids reach opposite poles and de-condense. Nuclear envelopes re-form and then cytokinesis occurs and yields two interphase daughter cells. The duration of mitosis means from prophase to telophase in this study.

**Chemical treatment and in vitro micronucleus assay**

Chemicals were purchased from Sigma-Aldrich (St., Louis). HeLa CENP B-GFP H2B-mCherry cells were maintained at 37℃ in a humidified atmosphere of 5% CO_2_ and 95% air. Exponentially growing cells were seeded at a density of 10^5^/well on 6-well plate and incubated for 24h and prepared for MN test using the cytokinesis-block technique.

The cells were treated with colcemid and actinomycin D with a final concentration of 25ng/mL and 15ng/mL, respectively, for 24h. The control cells were treated with DMSO. And then, K−MNi and K+MNi in the cells were scored by using fluorescent microscope. The number of micronucleated cells in at least 1000 binucleate (BN) cells was scored and the frequency of micronucleated cells per 1000 BN cells was calculated. The experiment was repeated for three times.

**DNA agarose gel electrophoresis for examining the DNA degradation in Hela Cells containing mainly K−MNs and K+MNs**

Exponentially growing cells were seeded at a density of 10^6^/dish (100 mm diameter) and incubated for 24h. Then the cells were treated with colcemid (a final concentration 25ng/mL) and actinomycin D (a final concentration 150 ng/mL and 15ng/mL), respectively, for 24h. The control cells were treated with DMSO. At the end of treatment, cells were centrifuged, and DNA was collected by using an EasyPure Genomic DNA Purification kit (Transgen Technology Co., Beijing) as followed according to the manufacturer’s protocol. The extracted DNA from each treatment was resuspended with 50μL deionized water. Approximately 1 μg of genomic DNA was loaded in each well, and DNA agarose gel electrophoresis was performed using 1.0% agarose (Sigma-Aldrich Corp.). The DNA was stained with Gel red (Sigma-Aldrich Corp.) and photographed under UV light.
