# Supplementary material for: The roles of kinetochore of micronucleus in mitosis of HeLa cells: a live cell imaging study
Source: Cancer Cell Int. 2019 Aug 2;19:206. doi: 10.1186/s12935-019-0917-8 (PMC6679434; doi:10.1186/s12935-019-0917-8)
Supplement: Supplementary file 2 — Additional file 2: Figure S1. Representative figure for duration of bipolar mitosis in a MN-free HeLa CENP B-GFP H2B-mCherry cell. Selected serial images (including mCherry, GFP and merged images) from time-lapse records showed four stage of mitosis. a. Prophase, the beginning of prophase is marked by the appearance of condensed chromosomes. b. Metaphase, the chromosomes align in the centre of the spindle, or the equatorial plate. c. Anaphase, the sister chromatids separate and move to opposite poles of the spindle. d. Telophase, the sister chromatids reach opposite poles and de-condense. [file 12935_2019_917_MOESM2_ESM.docx]

Figure 1


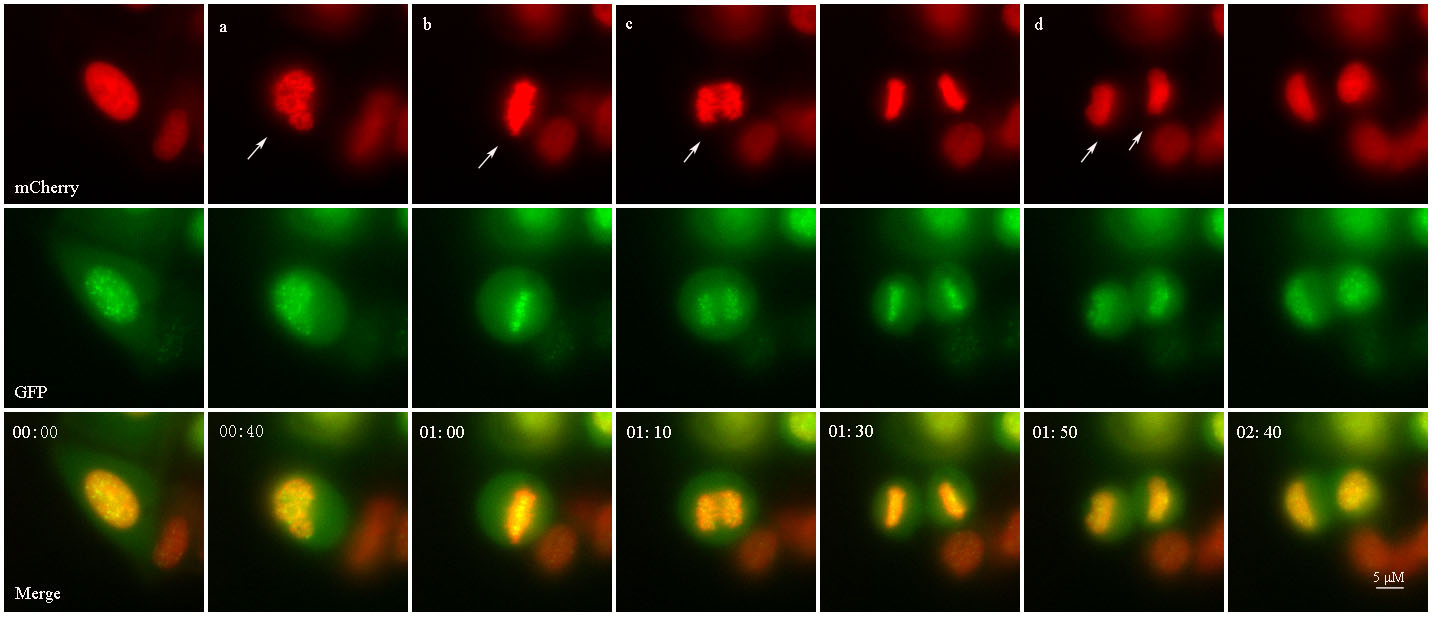


Figure 1 Representative figure for duration of bipolar mitosis in a MN-free HeLa CENP B-GFP H2B-mCherry cell. Selected serial images (including mCherry, GFP and merged images) from time-lapse records showed four stage of mitosis. a. Prophase, the beginning of prophase is marked by the appearance of condensed chromosomes. b. Metaphase, the chromosomes align in the centre of the spindle, or the equatorial plate. c. Anaphase, the sister chromatids separate and move to opposite poles of the spindle. d. Telophase, the sister chromatids reach opposite poles and de-condense.
