# Supplementary material for: The roles of kinetochore of micronucleus in mitosis of HeLa cells: a live cell imaging study
Source: Cancer Cell Int. 2019 Aug 2;19:206. doi: 10.1186/s12935-019-0917-8 (PMC6679434; doi:10.1186/s12935-019-0917-8)
Supplement: Supplementary file 3 — Additional file 3: Figure S2. Representative figures for apoptosis of a MN-free HeLa CENP B-GFP H2B-mCherry cell. Selected serial images (including mCherry, GFP and merged images) from time-lapse records showed apoptosis of a cell in mitosis. Arrows point to the initial cell nucleus, its pyknosis and Karyorrhexis. [file 12935_2019_917_MOESM3_ESM.docx]

Figure 2


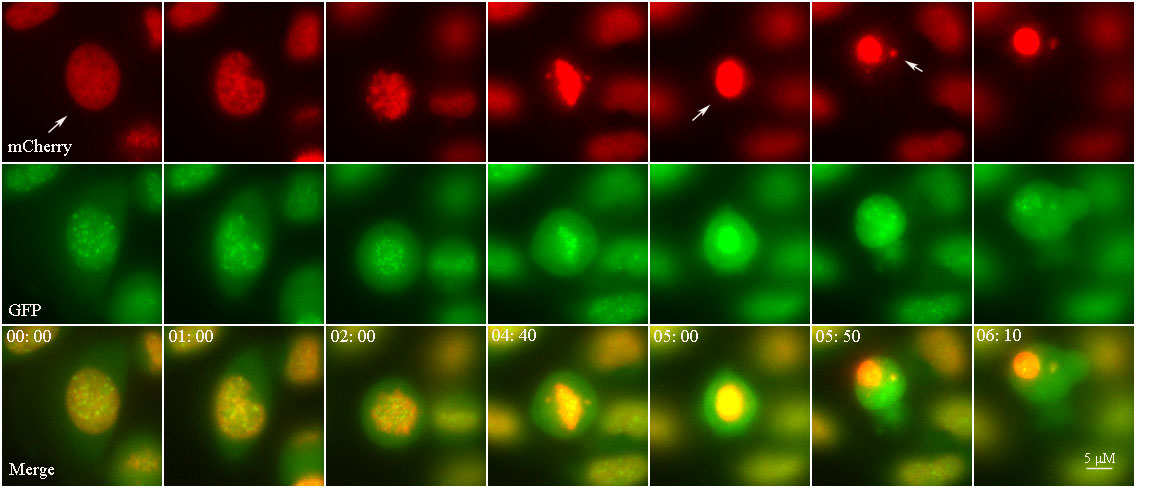


Figure 2 Representative figures for apoptosis of a MN-free HeLa CENP B-GFP H2B-mCherry cell. Selected serial images (including mCherry, GFP and merged images) from time-lapse records showed apoptosis of a cell in mitosis. Arrows point to the initial cell nucleus, its pyknosis and Karyorrhexis.
