# Supplementary material for: The roles of kinetochore of micronucleus in mitosis of HeLa cells: a live cell imaging study
Source: Cancer Cell Int. 2019 Aug 2;19:206. doi: 10.1186/s12935-019-0917-8 (PMC6679434; doi:10.1186/s12935-019-0917-8)
Supplement: Supplementary file 4 — Additional file 4: Table S1. Colcemid and actinomycin D induced K−MNi and K+MNi in HeLa CENP B-GFP H2B-mCherry cells. [file 12935_2019_917_MOESM4_ESM.docx]

**Table 1 Colcemid and actinomycin D induced K−MNi and K+MNi in HeLa CENP B-GFP H2B-mCherry cells**

|  | Concentration  (ng/mL) | Time of treatment (hour) | K−MNi/ binucleate cells (‰) | K+MNi/  binucleate cells (‰) | Total MNi/ binucleate cells (‰) |
| --- | --- | --- | --- | --- | --- |
| Control |  | 24 | 17.2± 2.31 | 15.6± 3.14 | 32.6± 2.82 |
| Colcemid | 25 | 24 | 21.6± 3.6 | 74.2±12.7* | 95.7±16.1* |
| Actinomycin D | 14 | 24 | 64.2±11.3* | 17.8± 4.31 | 81.8±15.4* |

(*p<0.05, compared with control. n=3.)

Induction of K−MNi and K+MNi were calculated and analyzed in the Table 1. Results showed that the proportional of K−MN is similar to K+MN in untreated cells (17.2 ± 2.31 ‰ VS 15.6± 3.14‰). Colcemid induced preferentially K+MNi and slightly increased K−MNi (74.2 ± 12.7‰ VS 21.6 ± 3.6‰). The frequency of overall MN in colcemid-treated cells is 2.94 times of the frequency of control. However, the frequency of K+MN in colcemid-treated cells is 4.76 times of that in control.

Contrary to colcemid, actinomycin D enhanced mainly K−MNi rather than K+MNi (64.2±11.3‰ VS 17.8± 4.31‰). Compared to control, the frequency of overall MN in Actinomycin D-treated cells shows a 2.51-fold increase, while the frequency of K−MN does a 3.73-fold increase. Subsequently, the classification of K−MN and K+MN can increase the specificity of the MN test.

The data suggested that HeLa CENP B-GFP H2B-mCherry cells can be applied in the in vitro micronucleus (MN) assay to investigate genotoxic effects of anticancer drugs. The study of the genotoxic effects of anticancer drugs contributed to understand the mechanism of their activity in the possible generation of secondary tumors.
