# Supplementary material for: The roles of kinetochore of micronucleus in mitosis of HeLa cells: a live cell imaging study
Source: Cancer Cell Int. 2019 Aug 2;19:206. doi: 10.1186/s12935-019-0917-8 (PMC6679434; doi:10.1186/s12935-019-0917-8)
Supplement: Supplementary file 5 — Additional file 5: Figure S3. DNA degradation was not obvious in Hela Cells containing mainly K−MNs and K+MNs. Cells were exposed to actinomycin D and colcemid and for 24 h, and DNA were isolated from each treatment for gel electrophoresis as described in “Methods” section. (1) 100 bp DNA ladder marker (Takara Corp.); (2) Control; (3) Cells treated with 150 ng/mL actinomycin D; (4) Cells treated with 15 ng/mL actinomycin D; (5) Cells treated with 25 ng/mL colcemid. Results suggested that there was no DNA degradation in control cells. DNA degradation was obvious in the high concentration of actinomycin D treatment (150 ng/mL), while slight DNA degradation occurred in the colcemid and low concentration actinomycin D (15 ng/mL) treatment cells. [file 12935_2019_917_MOESM5_ESM.docx]

Figure 3


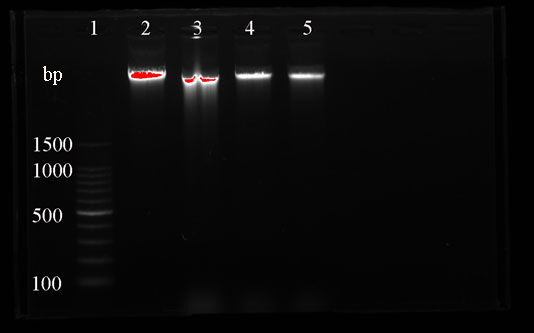


Figure 3 DNA degradation was not obvious in Hela Cells containing mainly K−MNs and K+MNs. Cells were exposed to actinomycin D and colcemid and for 24 h, and DNA were isolated from each treatment for gel electrophoresis as described in the Methods section. 1. 100bp DNA ladder marker (Takara Corp.); 2.Control; 3. Cells treated with 150 ng/mL actinomycin D; 4. Cells treated with 15ng/mL actinomycin D; 5. Cells treated with 25ng/mL colcemid. Results suggested that there was no DNA degradation in control cells. DNA degradation was obvious in the high concentration of actinomycin D treatment (150 ng/mL), while slight DNA degradation occurred in the colcemid and low concentration actinomycin D (15 ng/mL) treatment cells.
